# Assessing the policy utility of routine mortality statistics: a global classification of countries

**DOI:** 10.2471/BLT.22.289036

**Published:** 2023-12-01

**Authors:** Tim Adair, Lene Mikkelsen, Jessica Hooper, Azza Badr, Alan D Lopez

**Affiliations:** a The Nossal Institute for Global Health, Melbourne School of Population and Global Health, University of Melbourne, 32 Lincoln Square North, Carlton 3053, Victoria, Australia.; b LM Consulting, Tamborine Mountain, Australia.; c Manchester, England.; d Division of Data, Analytics and Delivery for Impact, World Health Organization, Geneva, Switzerland.

## Abstract

**Objective:**

To evaluate the utility and quality of death registration data across countries.

**Methods:**

We compiled routine death and cause of death statistics data from 2015–2019 from national authorities. We estimated completeness of death registration using the Adair-Lopez empirical method. The quality of cause of death data was assessed by evaluating the assignment of usable causes of death among people younger than 80 years. We grouped data into nine policy utility categories based on data availability, registration completeness and diagnostic precision.

**Findings:**

Of an estimated 55 million global deaths in 2019, 70% of deaths were registered across 156 countries, but only 52% had medically certified causes and 42% of deaths were assigned a usable cause. In 54 countries, which are mostly high-income, there is complete and high-quality mortality data. In a further 29 countries, located across different regions, death registration is complete, but cause of death data quality remains suboptimal. Additionally, 37 countries possess functional death registration systems with cause of death data of poor to moderate quality. In 30 countries, death registration ranges from limited to nascent completeness, accompanied by poor or unavailable cause of death data. Furthermore, 38 countries lack accessible data altogether.

**Conclusion:**

By implementing more proactive death notification processes, expanding the use of digitized data collection platforms, streamlining data compilation procedures and improving data quality assessment, governments could enhance the policy utility of mortality data. Encouraging the routine application of automated verbal autopsy methods is crucial for accurately determining the causes of deaths occurring at home.

## Résumé

**Objectif:**

Mesurer le degré d'utilité et la qualité de l'enregistrement de la mortalité selon les pays.

**Méthodes:**

Nous avons compilé les données statistiques habituelles relatives à la mortalité et aux causes de décès entre 2015 et 2019 en nous les procurant auprès des autorités nationales. Nous avons ensuite déterminé l'exhaustivité de l'enregistrement des décès à l'aide de la méthode empirique Adair–Lopez. La qualité des données sur les causes de décès a été mesurée en évaluant l'attribution des causes de décès utilisables parmi les personnes de moins de 80 ans. Enfin, nous avons regroupé les données en neuf catégories d'utilité politique en fonction de leur disponibilité, de l'exhaustivité de l'enregistrement et de la précision diagnostique.

**Résultats:**

Sur environ 55 millions de décès estimés à travers le monde en 2019, 70% d'entre eux ont été enregistrés dans 156 pays; en revanche, seuls 52% indiquaient des causes médicalement certifiées et 42% se sont vu assigner une cause utilisable. Dans 54 pays, principalement à revenu élevé, les données sur la mortalité sont complètes et de grande qualité. Dans 29 autres pays répartis entre différentes régions, l'enregistrement des décès est complet mais la qualité des données concernant les causes demeure insuffisante. En outre, 37 pays possèdent des systèmes fonctionnels d'enregistrement des décès, avec des données de qualité médiocre ou moyenne en ce qui concerne les causes. Dans 30 pays, l'enregistrement des décès présente une exhaustivité limitée à rudimentaire, et va de pair avec des données médiocres ou indisponibles quant aux causes de décès. Par ailleurs, 38 pays manquent totalement de données accessibles.

**Conclusion:**

En instaurant des processus plus proactifs de notification des décès, en étendant l'usage de plateformes de collecte de données numérisées, en simplifiant les procédures de regroupement de données et en améliorant l'évaluation de la qualité de ces données, les gouvernements devraient pouvoir accroître l'utilité politique des données de mortalité. Encourager l'application de méthodes d'autopsie verbale au quotidien est crucial pour déterminer avec précision les causes de décès survenus à domicile.

## Resumen

**Objetivo:**

Evaluar la utilidad y la calidad de los datos del registro de defunciones en diferentes países.

**Métodos:**

Se recopilaron datos estadísticos de rutina sobre defunciones y causas de muerte de 2015 a 2019 de las autoridades nacionales. Se estimó la completitud del registro de defunciones mediante el método empírico de Adair-Lopez. La calidad de los datos de causas de muerte se evaluó mediante la evaluación de la asignación de causas de muerte utilizables entre las personas menores de 80 años. Se agruparon los datos en nueve categorías de utilidad política basadas en la disponibilidad de datos, la completitud del registro y la precisión diagnóstica.

**Resultados:**

De los 55 millones de muertes en el mundo estimadas en 2019, el 70% de las muertes se registraron en 156 países; pero solo el 52% tenían causas médicamente certificadas y al 42% de las muertes se les asignó una causa utilizable. En 54 países, en su mayoría de ingresos altos, se dispone de datos de mortalidad completos y de alta calidad. En otros 29 países, situados en diferentes regiones, el registro de defunciones es completo, pero la calidad de los datos sobre la causa de muerte sigue siendo deficiente. Asimismo, 37 países cuentan con sistemas funcionales de registro de defunciones con datos sobre las causas de defunción de calidad deficiente a moderada. En 30 países, el registro de defunciones oscila entre un nivel de completitud limitado e incipiente, acompañado de datos sobre las causas de defunción deficientes o no disponibles. Además, 38 países carecen por completo de datos accesibles.

**Conclusión:**

Si los gobiernos implementaran procesos de notificación de defunciones más proactivos, ampliaran el uso de plataformas digitalizadas de recopilación de datos, racionalizaran los procedimientos de compilación de datos y mejoraran la evaluación de la calidad de los datos, podrían aumentar la utilidad política de los datos de mortalidad. Es fundamental fomentar la aplicación sistemática de métodos automatizados de autopsia verbal para determinar con exactitud las causas de las muertes que ocurren en el hogar.

## ملخص

**الغرض:**

تقييم فائدة بيانات تسجيل الوفيات وجودتها عبر الدول.

**الطريقة:**

قمنا بتجميع بيانات الإحصاءات الروتينية للوفيات وأسباب الوفاة للفترة من 2015 إلى 2019، من السلطات الوطنية. كذلك، قمنا بتقدير مدى اكتمال تسجيل الوفيات باستخدام طريقة أدير لوبيز التجريبية. تم تقييم جودة بيانات سبب الوفاة من خلال تقييم تحديد الأسباب المعقولة للوفاة بين الأشخاص الذين تقل أعمارهم عن 80 عامًا. قمنا بتجميع البيانات في تسع فئات لفوائد السياسة بناءً على مدى توفر البيانات، ومدى اكتمال التسجيل، ودقة التشخيص.

**النتائج:**

من بين ما يقدر بنحو 55 مليون حالة وفاة عالمية في عام 2019، تم تسجيل %70 من الوفيات في 156 دولة؛ لكن %52 فقط منها كانت لها أسباب معتمدة طبيًا، وتم تحديد سبب معقول في %42 من الوفيات. وفي 54 دولة، معظمها من الدول ذات الدخل المرتفع، تتوفر بيانات كاملة وعالية الجودة عن الوفيات. وفي 29 دولة أخرى، تقع عبر مناطق مختلفة، اكتمل تسجيل الوفيات، ولكن جودة بيانات أسباب الوفاة لا تزال دون المستوى الأمثل. وبالإضافة إلى ذلك، تمتلك 37 دولة أنظمة فعالة لتسجيل الوفيات، مع بيانات عن أسباب الوفاة ذات جودة رديئة إلى متوسطة. وفي 30 دولة، يتراوح تسجيل الوفيات من اكتمال محدود إلى اكتمال حديث العهد، مصحوبًا ببيانات رديئة أو غير متوفرة عن سبب الوفاة. علاوة على ذلك، تفتقر 38 دولة إلى البيانات التي يمكن الحصول عليها كليًا.

**الاستنتاج:**

من خلال تنفيذ عمليات أكثر استباقية للإبلاغ عن الوفاة، وتوسيع استخدام منصات جمع البيانات الرقمية، وتسهيل إجراءات تجميع البيانات، وتحسين تقييم جودة البيانات، يمكن للحكومات تعزيز الفائدة من سياسة بيانات الوفيات. يعد تشجيع التطبيق الروتيني لأساليب التشريح السردي الآلي، في غاية الاهمية لتحديد أسباب الوفيات التي تحدث في المنزل بدقة.

## 摘要

**目的:**

评估各国死亡登记数据的效用和质量。

**方法:**

我们从国家权威部门汇集了 2015 年至 2019 年期间的常规死亡和死因统计数据。我们使用 Adair-Lopez 经验法，对死亡登记数据的完整性进行了估计。通过评估 80 岁以下人群中可用死因的认定情况来评估死因数据的质量。我们根据数据可用性、登记完整性和诊断准确性将数据分为九个政策效用类别。

**结果:**

2019 年全球估计有 5,500 万人死亡，其中 70% 的死亡登记来自 156 个国家；但只有 52% 的死亡有死亡医学证明，42% 的死亡被认定为有可用死因。54 个国家（大多数为高收入国家）有完整和高质量的死亡率数据。还有 29 个国家（分布在不同区域）的死亡登记数据是完整的，但死因数据的质量未达最佳标准。同时， 37 个国家拥有实用的死亡登记系统，但死因数据质量较差或中等。30 个国家的死亡登记数据的完整性不足或刚开始进行登记，同时其死因数据不足或不可用。此外，38 个国家完全没有可使用的数据。

**结论:**

通过实施更主动的死亡通知程序、扩大数字化数据收集平台的使用、简化数据编制程序和改进数据质量评估，政府可以提高死亡率数据的政策效用。推进自动死因推断方法的常规使用对于准确确定发生在家中的死亡的原因至关重要。

## Резюме

**Цель:**

Оценить пользу и качество данных регистрации смерти в разных странах.

**Методы:**

От национальных органов власти были собраны данные регулярной статистики смертности и причин смерти за 2015–2019 гг. Полнота регистрации случаев смерти оценивалась с использованием эмпирического метода Адера – Лопеса. Качество данных о причинах смерти оценивалось по распределению возможных причин смерти среди лиц моложе 80 лет. Данные были распределены по девяти категориям полезности с программно-нормативной точки зрения в зависимости от доступности, полноты регистрации и точности диагностики.

**Результаты:**

Примерно из 55 млн случаев смерти в мире в 2019 году 70% смертей были зарегистрированы в 156 странах, однако только 52% из них были подтверждены с медицинской точки зрения, а в 42% случаев была установлена возможная причина смерти. В 54 странах, в основном с высоким уровнем доходов, доступны полные и качественные данные о смертности. Еще в 29 странах, расположенных в различных регионах, регистрация случаев смерти выполняется, однако качество данных о причинах смерти остается неудовлетворительным. Кроме того, в 37 странах действуют функциональные системы регистрации случаев смерти, в которых данные о причинах смерти характеризуются низким или умеренным качеством. В 30 странах регистрация случаев смерти варьируется от ограниченной до начальной полноты и сопровождается скудными или отсутствующими данными о причинах смерти. Кроме того, в 38 странах доступные данные отсутствуют совсем.

**Вывод:**

Благодаря внедрению более активных процессов уведомления о случаях смерти, расширению использования цифровых платформ сбора данных, оптимизации методов сбора данных и улучшению оценки их качества органы власти могут повысить уровень пользы данных о смертности. Поощрение применения стандартных методов автоматизированной вербальной аутопсии имеет решающее значение для точного определения причин смерти на дому.

## Introduction

Health planning and policy evaluation requires reliable, detailed and timely mortality statistics and cause of death data. Functional civil registration and vital statistics systems ensure the continuous registration and certification of all deaths in a population.^1^ However, in many countries, the statistics on deaths and causes of death produced by civil registration and vital statistics systems are inaccurate and otherwise of limited use for public policy.^2^ Even when proper registration and certification of deaths occur, the information is not always compiled into routine statistics to guide government policy. Gaps in mortality data are often filled by surveys and censuses despite the fact that they are not a timely and routine source of mortality statistics.^3^ In many low- and middle-income countries cause of death data are only collected for the small proportion of deaths that occur in hospitals. Moreover, the diagnostic accuracy and policy utility of such statistics are limited by insufficient training in correct medical certification procedures and/or incorrect coding practices.^4^ Approximately 60% of deaths occur outside of hospitals in low- and middle-income countries.^5^ Data on these deaths can be unreliable because the cause is often ascertained by non-medical, untrained personnel, with or without the use of validated verbal autopsy methods. 

Civil registration and vital statistics are designed to generate continuous, disaggregated, timely and accurate data on deaths and causes of death that can be used as a source of health intelligence for governments. Mortality data that is categorized by both age and gender can be used to compute essential population health metrics like life expectancy, and to monitor a nation's progress through the epidemiological transition. Reliable measurements of excess mortality during pandemics or from mortality shocks such as natural disasters or civil conflict are critical for decision-making. In addition, civil registration and vital statistics systems facilitate calculation of mortality indicators for subnational populations; in turn enabling a more granular understanding of differentials in mortality risk. Yet, despite these multiple uses, death registration data are often underused largely because of concerns about their accuracy and potential biases.^6^

Similarly, reliable and timely statistics on causes of death are fundamental for tracking trends in the leading causes of premature mortality; for underpinning assessments of the impact of health intervention strategies and policies; and are a prerequisite for monitoring progress for seven out of the 17 sustainable development goals.^7^^,^^8^

Previous global assessments of the availability and quality of mortality statistics generated by civil registration and vital statistics systems are limited to a single source, such as the World Health Organization (WHO) Mortality Database, and rely on complex transformations of the underlying data; or are now several years out of date.^1^^,^^2^^,^^9^^,^^10^ A recent assessment of mortality data produced by the Global Burden of Disease (GBD) Study focused on the utility of vital registration for measuring national and subnational burden of disease; however they failed to identify common challenges in countries at different levels of development of statistical systems, and often censored data points judged to be outliers, introducing bias into the results.^11^

Here we present a systematic evaluation of the completeness and quality of death and cause of death statistics for all publicly available sources of data where deaths were either registered or otherwise known to a government. We then use these results to classify country vital registration systems into categories that represent their utility for guiding public policy in countries. Based on these scores, we then propose a prioritized set of feasible, tested system interventions that can assist countries to derive maximum policy benefit from their administrative data systems.

## Methods

We used death statistics generated from routine death registration or reporting systems designed to count all events within a specific jurisdiction, including hospital death reporting systems. We define these statistics as registered or reported deaths known to some level of government authority; hereafter we refer to them as registered deaths. Data sources were identified from an exhaustive search, and were only considered if they were compiled by national authorities and publicly available either through national reports (e.g. vital statistics reports); international databases which include only statistics notified by national authorities; national civil registration and vital statistics system assessments endorsed by governments; or data otherwise made available to the authors by relevant government agencies.^10^^,^^12^^,^^13^ These sources exclude mortality and cause of death statistics collected from Sample Registration Systems; Health and Demographic Surveillance System sites; and other surveys and censuses where the primary intent was not formal death registration. We used the WHO Mortality Database for cause of death data for the majority of countries, and country-provided data in some instances.^10^ A full list of data sources is provided in the online repository.^14^ We only considered data from 2015–2019 as indicative of an operational vital registration system, and we did not consider timeliness any further.

We evaluated the policy utility of vital registration data, based on the completeness of death registration and quality of cause of death data. Completeness was calculated for registered or reported deaths as the percentage of the estimated total number of deaths, measured where possible by year of occurrence using the Adair-Lopez empirical completeness method.^15^ This method estimates completeness of death registration using a statistical model with covariates of the registered crude death rate, and incorporates variables representing the drivers of the true crude death rate such as: (i) the under-five mortality rate to represent mortality level; (ii) the percentage of the population aged 65 years or older to represent population age structure; (iii) the completeness of under-five death registration or reporting; (iv) calendar year; and (v) country random effects.^15^ Such a method also ensures comparability by applying a common approach to calculating completeness for all data.

For countries where there is either high human immunodeficiency virus mortality or conflict-driven mortality, we used the United Nations (UN) World Population Prospects^16^ estimated deaths to calculate completeness. The empirical completeness method is less reliable in such settings given the assumptions of the model; these countries are indicated in the online repository.^14^ UN estimates are predominantly derived from adult mortality calculated from registered deaths adjusted for incompleteness, and child and adult mortality estimates from censuses and surveys, which are entered into model life tables.^16^ We present completeness estimates in bands of five percentage points to account for uncertainty in estimation. We estimated global completeness of death registration by using the most recent completeness estimate for each country based on available data for the period 2015–2019; as applied to UN Population Division estimates of deaths in 2019.^16^

We included all cause of death data where the underlying cause was ascertained from the International certificate of medical cause of death (medically certified cause of death), subsequently coded according to the rules and procedures of the *International statistical classification of diseases and related health problems*, 10th revision (ICD-10), 2016 version.^17^ Garbage codes were classified into four different levels of severity (very high, high, medium and low) according to their potential effect on policy deliberations (online repository).^14^^,^^18^ Using this classification, we calculated the percentage of deaths with a useable cause; that is, the percentage of estimated total deaths that have a medically certified cause of death and for which the assigned cause was not classified as a garbage code of very high, high or medium severity.

Countries with a high proportion of deaths at older ages (older than 80 years) tend to have a higher proportion of garbage codes in their cause of death data because accurately diagnosing underlying cause of death at older ages is increasingly difficult due to higher likelihood of multiple morbidities. We therefore only considered cause of death data for those individuals younger than 80 years, to ascertain the extent of garbage codes in the data. 

For 46 countries the distribution of garbage codes by severity could not be calculated because ICD-10 data were not publicly available. For these, we predicted the useable fraction of causes of death for individuals younger than 80 years based on a linear regression model using death registration completeness; GBD super-region; sociodemographic index; and universal health coverage as covariates (online repository).^14^ Usability calculations and estimates are also presented in bands of five percentage points to account for uncertainty in the completeness estimation method. Estimating the fraction of useable causes for these 46 countries enabled their inclusion in further analyses of civil registration and vital statistics system performance. We did not include country-level estimated usability percentages in global or regional calculations of usability.

To better target interventions and identify challenges in mortality statistics, the usability of countries’ data was classified by the completeness of death registration and the quality of cause of death data, determined by the percentage of deaths in individuals younger than 80 years with a usable code. The criteria for categorizing mortality data from national vital registration systems are displayed in Table 1. Countries with 95%–100% death registration are labelled as complete since the small percentage of unrecorded deaths would unlikely influence policy decisions. Those with 75%–94% registration are termed functional; they record enough deaths to demonstrate that the system is operational, but many deaths go unrecorded. Countries with 25%–74% registration have limited systems. Despite being operational, a significant portion of deaths remain unrecorded, reducing the policy value of the data. Nascent registration indicates very few recorded deaths, highlighting a need to enhance basic system components. High-quality cause of death data means 80% or more deaths in people younger than 80 years have a usable cause, marking them as fit-for-purpose. Data with 60%–79% usable causes are moderate quality, suitable for policy discussions but requiring caution. Data with less than 60% usable causes are of poor quality. In many such countries, non-medical staff often determine causes, sometimes using verbal autopsy. While these classifications and thresholds might seem arbitrary, they offer a foundation to prioritize interventions for enhancing the usefulness of regular mortality data.

**Table 1:** Criteria for the classification of mortality data from national vital registration systems

Categories of mortality data	%	
	Registration completeness	Deaths assigned a useable cause of death^a^
Complete registration and high quality cause of death	95–100	80–100
Complete registration and moderate quality cause of death	95–100	60–79
Complete registration and poor quality cause of death	95–100	< 60
Functional registration and high quality cause of death	75–94	80–100
Functional registration and moderate quality cause of death	75–94	60–79
Functional registration and poor quality cause of death	75–94	< 60
Limited registration and poor quality cause of death	25–74	< 60
Nascent death registration and cause of death data	> 0 but < 25	> 0 but ≤ 10
Data not available	N/A	N/A

^a^ Of deaths reported for those younger than 80 years.

Note: We classified countries where the most recent data were collected before 2015 as having no data available.

## Results

Of the more than 55 million deaths estimated to have occurred worldwide in 2019, 70% were captured and reported by civil registration and vital statistics systems in 156 countries. Just over half (52%) had a medically certified cause of death, while only 42% of deaths in people younger than 80 years were assigned a useable cause of death; the remainder either being attributed to a garbage cause of high, medium or low severity; or where the medically certified cause of death contained insufficient detail to assess quality (Table 2). The African Region only registers a small minority of deaths (15% of estimated total deaths in that region) and even less with a medically certified cause of death (8%). Eastern Mediterranean (50%) and South-East Asia Regions (69%) each have moderate levels of death registration, but far lower medically certified cause of death completeness (28% and 17%, respectively) and cause of death usability (15% and 3%, respectively). Other regions have higher death registration completeness, with cause of death usability being highest in the European Region at 82%.

**Table 2:** Completeness and quality of death registration and cause of death statistics by region; 2019

WHO Region	%		
	Deaths registered	Deaths with medically certified cause of death	Deaths with useable cause^a^
African Region	15	8	4
Region of the Americas	95	90	77
South-East Asia Region	69	17	4
European Region	99	98	82
Eastern Mediterranean Region	50	28	17
Western Pacific Region	82	72	70
**Global**	**70**	**52**	**42**

WHO: World Health Organization.

^a^ Includes deaths reported for those younger than 80 years.

**Fig. 1:**
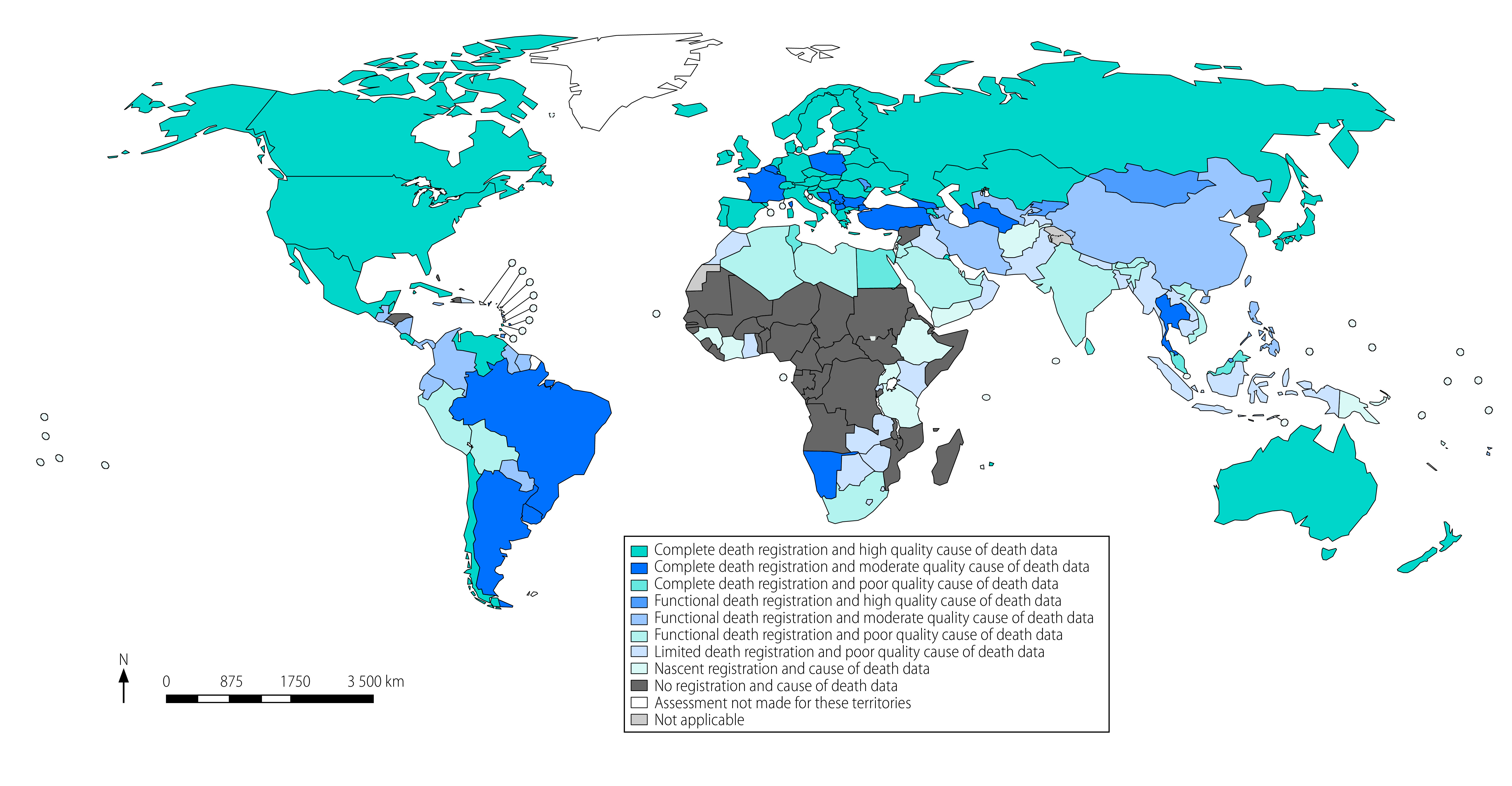
Estimated completeness and quality of mortality and cause of death data for WHO Member States, 2019

The distribution of all 194 WHO Member States according to the two primary measures of data usability is summarized in Table 3 and presented by country in Fig. 1 and in the online repository.^14^ More than half (83) of the 156 reporting countries had death registration levels of at least 95%; and of these, about 54 had systems that provided a useable cause of death for at least 80% of deaths among people younger than 80 years. Death registration systems in these 54 countries can be considered as complete, and of high quality, accounting for about one-quarter of global deaths, with most of them located in the Region of the Americas, the European Region and the five-high income countries of the Western Pacific Region (Fig. 1). A further 29 geographically diverse countries have systems that successfully capture all or most deaths; but only 60%–80% of deaths that occur are assigned a useable cause in 20 of these countries, falling to less than 60% in a further nine countries. Of the remainder, more than half (43 countries) had systems that were at least 75% complete, and in some cases close to 95% complete; but where cause of death data quality was assessed as being predominantly moderate (20 countries) or poor (17 countries).^18^

**Table 3:** Distribution of WHO Member States according to completeness, availability and quality of cause of death, 2019

Categories of mortality data	No. of countries	Estimated % of global deaths
Complete registration and high quality cause of death	54	23
Complete registration and moderate quality cause of death	20	7
Complete registration and poor quality cause of death	9	2
Functional registration and high quality cause of death	6	< 1
Functional registration and moderate quality cause of death	20	21
Functional registration and poor quality cause of death	17	20
Limited registration and poor quality cause of death	21	11
Nascent death registration and cause of death data	9	4
Data not available	38	11
**Total**	**194**	**100**

WHO: World Health Organization.

Note: The definition of each category is presented in Table 1.

WHO: World Health Organization.

A geographically diverse group of 21 countries account for about one tenth of global deaths and have vital registration systems that provide limited policy benefit; typically capturing between 25%–74% of deaths but with useable fractions well below 60%. We identified data for nine countries where data completeness and quality are so low that we have classified the data systems as nascent. For 38 countries, we could find no vital registration data on mortality, or the most recent data was from before 2015. Likely, these 38 countries have some form of mortality reporting system established and operated by a government; however, the data were unidentifiable using our search strategy.

## Discussion

In 2019, 70% of global deaths were reported to a government agency, suggesting a potential improvement from the 59% global death registration rate indicated by the GBD Study in 2015.^19^ However, less than half of deaths among individuals younger than 80 years received a cause of death usable for public health planning. While nearly half of WHO Member States provided comprehensive mortality data, in 38 countries no data could be identified from 2015 to 2019.

We found that for approximately half of deaths, the cause of death is not determined from a medically certified cause of death, and when they are, a significant portion either gets attributed to a cause of no public health utility or lacks specific details. Addressing this problem requires proper physician training to ensure accurate death certification. Proper cause of death determination would also have a positive impact on reducing misdiagnoses among deaths younger than 80 years, where the cause is useable but not necessarily correct. Several studies of diagnostic accuracy in low- and middle-income countries have reported misdiagnosis levels that are typically around 30% of useable causes; further reducing the policy value of routine vital statistics.^4^^,^^20^^–^^22^

We classified countries into nine categories of data usability based on a joint assessment of completeness and quality, quantified by the fraction of garbage codes found in the data. Countries that have complete registration also tend to have higher quality cause of death data. These were mostly high-income countries, and civil registration and vital statistics improvement strategies in these populations should focus on improving diagnostic practices among physicians to reduce garbage coding, especially for deaths in individuals younger than 80 years. Most countries with functional, but still incomplete registration also have substantial challenges with cause of death data quality. Countries in this category tended to be more geographically diverse. In countries where mortality data is nascent or exhibits low completeness and poor certification quality, enhancing vital statistics systems should be a priority. These efforts should include capacity-building and training in data analysis and dissemination as key strategies for reliably monitoring progress of forthcoming global health initiatives.

The coronavirus disease 2019 (COVID-19) pandemic highlighted the poor state of death registration systems in many countries, with most low- and middle-income countries being unable to generate timely and reliable data on excess mortality during the pandemic. Globally, only 73 of 194 countries produced full national data in 2020 and 2021 to enable calculation of excess mortality; most of them high-income countries.^23^ Measuring excess mortality reliably becomes challenging when registration is incomplete; distinguishing actual mortality increases from the pandemic's effects on the death registration system is a complex task.^24^^,^^25^ In many countries during the pandemic, death registration systems were unable to function as normal due to the impact of movement restrictions, a reliance on paper-based registration, and as a consequence of COVID-induced mortality.^26^ In Peru, for example, health services were overwhelmed due to a surge in deaths which meant that normal registration processes were severely disrupted; while in Ghana the operations of some civil registration offices were adversely affected by movement restrictions.^26^^–^^28^ The pandemic highlighted the need for governments to have reliable mortality statistics, which resulted in increased use of online registration, which has improved efficiency with attendant longer-term benefits for improved global death registration.^27^^–^^29^

Improving civil registration and vital statistics systems is a significant, organizational, technical and financial challenge for many countries. Countries seeking to improve their civil registration and vital statistics systems can benefit from the *WHO Civil registration and vital statistics strategic implementation plan 2021–2025*. This comprehensive platform enables countries to prioritize interventions tailored to their needs, emphasizing strengthened intersectoral collaboration and governance for vital statistics.^30^ This plan offers many feasible options for improving death registration and diagnosis based on recent methodological research and experience with practical implementation in countries. Specifically, this cumulated experience suggests that governments can work to improve the completeness of registered deaths by focusing on more active death notification processes for deaths occurring outside hospitals; on innovative application of digitized data collection platforms; on improving the national compilation of data; and on implementing processes to assess data quality.^6^ Concurrently, there should be accelerated efforts to increase use of the International medical certificate of cause of death (with appropriate training of physicians to reduce deaths with a garbage cause), and to fill the cause of death data gap for non-hospital deaths with routine, validated automated verbal autopsy methods.^21^^,^^31^ Training of physicians, including in medical school curricula, should be a priority in countries with complete registration but more than 20% of garbage codes, using the effective training methods and tools available.^21^^,^^22^ The introduction of ICD-11 will present additional challenges for its implementation into death registration systems; reinforcing the need for accelerated efforts to train doctors in correct medical certification procedures for deaths; and coders in the application of ICD-11 coding rules. 

Monitoring the impact of civil registration and vital systems interventions through periodic assessment of data completeness and quality can now easily be performed for subnational populations using established data quality assessment software, such as ANACONDA (co-developed by the University of Melbourne, Melbourne, Australia and the Swiss Tropical and Public Health Institute, University of Basel, Basel, Switzerland).^32^ The composition of the package of interventions that should be prioritized under the WHO *Civil registration and vital statistics strategic implementation plan 2021–2025* will vary according to the needs and capacity of countries, but is likely to be similar for all countries in each of the categories that we have identified.^30^

The persistent application of these strategies is likely to bring about significant improvements in the availability and policy utility of mortality statistics. However, significant improvements in completeness of death registration may take some time, possibly a decade or longer in some countries. In the interim, established demographic methods applied to child survival, sibling survival and household death data available from surveys or censuses offer an alternative means for generating essential health intelligence on levels and patterns of mortality required to inform health planning.^33^ The policy utility of this information will be substantially enhanced by the application of automated verbal autopsy methods to a representative sample of deaths collected in surveys or censuses to provide cause of death data, as has been demonstrated elsewhere.^34^

Our study has some limitations, most notably the omission of recent vital statistics that are undoubtedly being produced in several low- and middle-income countries but are not readily accessible; as well as more disaggregated analysis of data quality at a subnational level where data are likely to be most relevant for planning. Estimates of death registration completeness are also subject to accuracy in the method used, either the empirical completeness method, or GBD or world population prospects estimated deaths. We used a standardized approach to estimate completeness rather than relying on country-derived estimates that would vary by country and lack suitability, which would reduce their intercountry comparability. We also did not assess completeness of death registration during the COVID-19 pandemic, in part because data for the pandemic years were not yet widely available, but also because of the abnormal registration environment that prevailed in most countries due to national responses to the pandemic, including movement restrictions. We were also unable to assess the role of public versus private facilities in reporting deaths. There is little available information on the relative completeness or accuracy of death data from different types of facilities, or even whether they were recorded in private or public institutions.

Vital statistics on deaths and causes of death are the cornerstone of any country’s health information system, and the findings of this study should guide national and global action to improve them. Better data will allow countries to make better decisions leading to enhanced population health.

## References

[1] Phillips DE, Lozano R, Naghavi M, Atkinson C, Gonzalez-Medina D, Mikkelsen L, et al. A composite metric for assessing data on mortality and causes of death: the vital statistics performance index. Popul Health Metr. 2014 May 14;12(1):14. 10.1186/1478-7954-12-14 24982595 PMC4060759

[2] Mikkelsen L, Phillips DE, AbouZahr C, Setel PW, de Savigny D, Lozano R, et al. A global assessment of civil registration and vital statistics systems: monitoring data quality and progress. Lancet. 2015 Oct 3;386(10001):1395–406. 10.1016/S0140-6736(15)60171-4 25971218

[3] Mahapatra P, Shibuya K, Lopez AD, Coullare F, Notzon FC, Rao C, et al.; Civil registration systems and vital statistics: successes and missed opportunities. Lancet. 2007 Nov 10;370(9599):1653–63. 10.1016/S0140-6736(07)61308-7 18029006

[4] Rampatige R, Mikkelsen L, Hernandez B, Riley I, Lopez AD. Systematic review of statistics on causes of deaths in hospitals: strengthening the evidence for policy-makers. Bull World Health Organ. 2014 Nov 1;92(11):807–16. 10.2471/BLT.14.137935 25378742 PMC4221770

[5] Adair T. Who dies where? Estimating the percentage of deaths that occur at home. BMJ Glob Health. 2021 Sep;6(9):e006766. 10.1136/bmjgh-2021-006766 34479953 PMC8420738

[6] Adair T, Rajasekhar M, Bo KS, Hart J, Kwa V, Mukut MAA, et al. Where there is no hospital: improving the notification of community deaths. BMC Med. 2020 Mar 9;18(1):65. 10.1186/s12916-020-01524-x 32146904 PMC7061465

[7] Hart JD, Sorchik R, Bo KS, Chowdhury HR, Gamage S, Joshi R, et al. Improving medical certification of cause of death: effective strategies and approaches based on experiences from the Data for Health Initiative. BMC Med. 2020 Mar 9;18(1):74. 10.1186/s12916-020-01519-8 32146900 PMC7061467

[8] Richards N, Sorchik R, Brolan C. Why the sustainable development goal agenda needs strong civil registration and vital statistics systems. Carlton: University of Melbourne, Civil Registration and Vital Statistics Improvement, Bloomberg Philanthropies Data for Health Initiative; 2018.

[9] Mathers CD, Fat DM, Inoue M, Rao C, Lopez AD. Counting the dead and what they died from: an assessment of the global status of cause of death data. Bull World Health Organ. 2005 Mar;83(3):171–7. 15798840 PMC2624200

[10] WHO mortality database. Geneva: World Health Organization; 2021. Available from: https://www.who.int/data/data-collection-tools/who-mortality-database [cited 2023 Nov 2].

[11] GBD 2019 Demographics Collaborators. Global age-sex-specific fertility, mortality, healthy life expectancy (HALE), and population estimates in 204 countries and territories, 1950-2019: a comprehensive demographic analysis for the Global Burden of Disease Study 2019. Lancet. 2020 Oct 17;396(10258):1160–203. 10.1016/S0140-6736(20)30977-6 33069325 PMC7566045

[12] Global burden of disease deaths database. Seattle: The Institute For Health Metrics And Evaluation; 2020. Available from: https://ghdx.healthdata.org/gbd-2019/data-input-sources [cited 2023 Nov 2].

[13] Population and vital statistics report, live births, deaths, and infant deaths. New York: United Nations; 2021.

[14] Adair T, Mikkelsen L, Hooper J, Badr A, Lopez AD. Policy utility of routine mortality statistics: a global classification of countries [online repository]. London: Figshare; 2023. 10.26188/24457039 PMC10680110 38046370

[15] Adair T, Lopez AD. Estimating the completeness of death registration: An empirical method. PLoS One. 2018 May 30;13(5):e0197047. 10.1371/journal.pone.0197047 29847573 PMC5976169

[16] World population prospects: the 2019 revision. New York: United Nations; 2019.

[17] International statistical classification of diseases and related health problems 10th revision (ICD-10): 2016 version. Geneva: World Health Organization; 2016.

[18] Naghavi M, Richards N, Chowdhury H, Eynstone-Hinkins J, Franca E, Hegnauer M, et al. Improving the quality of cause of death data for public health policy: are all ‘garbage’ codes equally problematic? BMC Med. 2020 Mar 9;18(1):55. 10.1186/s12916-020-01525-w 32146899 PMC7061466

[19] Dicker D, Nguyen G, Abate D, Abate KH, Abay SM, Abbafati C, et al. GBD 2017 Mortality Collaborators. Global, regional, and national age-sex-specific mortality and life expectancy, 1950-2017: a systematic analysis for the Global Burden of Disease Study 2017. Lancet. 2018 Nov 10;392(10159):1684–735. 10.1016/S0140-6736(18)31891-9 30496102 PMC6227504

[20] Chen L, Xia T, Yuan ZA, Rampatige R, Chen J, Li H, et al. Are cause of death data for Shanghai fit for purpose? A retrospective study of medical records. BMJ Open. 2022 Feb 15;12(2):e046185. 10.1136/bmjopen-2020-046185 35168960 PMC8852669

[21] Gamage USH, Mahesh PKB, Schnall J, Mikkelsen L, Hart JD, Chowdhury H, et al. Effectiveness of training interventions to improve quality of medical certification of cause of death: systematic review and meta-analysis. BMC Med. 2020 Dec 11;18(1):384. 10.1186/s12916-020-01840-2 33302931 PMC7728523

[22] Lucero M, Riley ID, Hazard RH, Sanvictores D, Tallo V, Dumaluan DGM, et al. Assessing the quality of medical death certification: a case study of concordance between national statistics and results from a medical record review in a regional hospital in the Philippines. Popul Health Metr. 2018 Dec 29;16(1):23. 10.1186/s12963-018-0178-0 30594186 PMC6311069

[23] Methods for estimating the excess mortality associated with the COVID-19 pandemic. Geneva; World Health Organization; 2022.

[24] Adair T, Hudson S, Lopez AD. Approaches and methods for estimating excess deaths due to COVID-19: civil registration and vital systems best practice and advocacy. Melbourne: Bloomberg philanthropies data for health initiative; 2021.

[25] Lima EEC, Vilela EA, Peralta A, Rocha M, Queiroz BL, Gonzaga MR, et al. Investigating regional excess mortality during 2020 COVID-19 pandemic in selected Latin American countries. Genus. 2021;77(1):30. 10.1186/s41118-021-00139-1 34744175 PMC8564791

[26] AbouZahr C, Bratschi MW, Cercone E, Mangharam A, Savigny D, Dincu I, et al. The COVID-19 pandemic: effects on civil registration of births and deaths and on availability and utility of vital events data. Am J Public Health. 2021 Jun;111(6):1123–31. 10.2105/AJPH.2021.306203 33856881 PMC8101592

[27] Silva-Valencia J, Adair T, Hart J, Meza G, Vargas Herrera J. How has COVID-19 impacted the civil registration and vital statistics system in Loreto, Perú? Evidence using process mapping and qualitative analysis. BMJ Open. 2021 Nov 19;11(11):e055024. 10.1136/bmjopen-2021-055024 34799366 PMC8609502

[28] United Nations, Department of Economic and Social Affairs, Statistics Division. Legal Identity Agenda. 2020 impact of COVID-19. 2020. Available from: https://unstats.un.org/legal-identity-agenda/covid-19 [cited 2023 Nov 2].

[29] Kelly M, Mathenge G, Rao C. Lessons learnt and pathways forward for national civil registration and vital statistics systems after the COVID-19 pandemic. J Epidemiol Glob Health. 2021 Sep;11(3):262–5. Error! Hyperlink reference not valid. 10.2991/jegh.k.210531.001 34270182 PMC8435876

[30] WHO civil registration and vital statistics strategic implementation plan 2021–2025. Geneva: World Health Organization; 2021.

[31] Hazard RH, Buddhika MPK, Hart JD, Chowdhury HR, Firth S, Joshi R, et al. Automated verbal autopsy: from research to routine use in civil registration and vital statistics systems. BMC Med. 2020 Mar 9;18(1):60. 10.1186/s12916-020-01520-1 32146903 PMC7061477

[R32] 32. Mikkelsen L, Moesgaard K, Hegnauer M, Lopez AD. ANACONDA: a new tool to improve mortality and cause of death data. BMC Med. 2020 Mar 9;18(1):61. 10.1186/s12916-020-01521-0 32146907 PMC7061487

[33] Hill K, Lopez AD, Shibuya K, Jha P. Monitoring of Vital Events (MoVE). Interim measures for meeting needs for health sector data: births, deaths, and causes of death. Lancet. 2007 Nov;370(9600):1726–35. 10.1016/S0140-6736(07)61309-9 18029005

[34] Mozambique National Institute of Statistics, United States Census Bureau, MEASURE Evaluation, United States Centers for Disease Control and Prevention. Mortality in Mozambique: results from a 2007–2008 post-census mortality survey. Chapel Hill: MEASURE Evaluation; 2012.

